# Supplementary material for: Ultrafast Nonlinear Dynamics of Indium Tin Oxide Nanocrystals Probed via Fieldoscopy
Source: Adv Sci (Weinh). 2025 Nov 6;13(10):e16818. doi: 10.1002/advs.202516818 (PMC12915141; doi:10.1002/advs.202516818)
Supplement: Supplementary file 1 — Supporting Information [file ADVS-13-e16818-s001.pdf]

Ultrafast Nonlinear Dynamics of Indium Tin Oxide Nanocrystals Probed via Fieldoscopy

*Andreas Herbst Anchit Srivastava Kilian Scheffter Soyeon Jun Steffen Gommel Luca Rebecchi Sidharth Kuriyil Andrea Rubino Nicolò Petrini Ilka Kriegel Hanieh Fattahi**

Supplementary Information

Calculation of the Field Response

The measured field response E_S shown in the main text is a convolution between the excitation pulse E_R and the complex time domain response of the sample $R(t)$:

$$E_S(t) = (E_R * R)(t). \tag{1}$$

To gain access to the complex response of the sample in the frequency domain, the convolution theorem can be employed

$$\mathcal{F}(E_S)(\omega) = \mathcal{F}(E_R)(\omega) \cdot \mathcal{F}(R)(\omega) = \hat{E}_R(\omega) \cdot \hat{R}(\omega). \tag{2}$$

The complex transmission $\hat{R}(\omega)$ can be extracted via division of the two complex spectra measured with and without the sample

$$\hat{R}(\omega) = \frac{\hat{E}_S(\omega)}{\hat{E}_R(\omega)} = |\hat{R}(\omega)| \cdot e^{i\cdot\phi_R(\omega)} \tag{3}$$

with full amplitude and phase information. The amplitude of the attenuation in Figure 2 d in the main text is calculated via $A(\omega) = 1-|\hat{R}(\omega)|^2$ to be comparable with intensity-based techniques. This is mathematically equivalent to $\Delta I/I_R$, which is a commonly used term:

$$\frac{\Delta I}{I_R} = \frac{I_R - I_S}{I_R} \propto \frac{|\hat{E}_R|^2 - |\hat{E}_S|^2}{|\hat{E}_R|^2} = 1 - |\hat{R}(\omega)|^2 \tag{4}$$

while still maintaining the phase information contained in the argument of the complex response $\phi = arg(\hat{R}(\omega))$ shown in Figure 2 d. The conversion of $\hat{R}(\omega)$ back into the time domain (de-convolution) is highly sensitive to noise, so this technique is limited to spectral bands with a sufficiently high signal-to-noise ratio (SNR) of the reference at best. For robust analysis of the interaction between light and matter directly in the time domain, the following approach was used:

$$E_{Response}(t) := \frac{E_R(t) - E_S(t-\tau)}{\max(|E_R(t)|)} \tag{5}$$

$$\implies \mathcal{F}(E_{Response})(\omega) \propto \hat{E}_R(\omega) - \hat{E}_S(\omega) \cdot e^{-i\omega\tau} = \hat{E}_R(\omega) \cdot (1 - \hat{R}(\omega) \cdot e^{-i\omega\tau}). \tag{6}$$

The $E_{Response}$ carries information on the phase and amplitude changes of the excitation pulse after transmission from the sample; however, it is not mathematically equivalent to the complex material response R, as it is convoluted with $E_R(\omega)$. The Fourier transformation of $E_{Response}(t)$ presents a virtual interferometric measurement, which contains information on both absorption and dephasing relative to the known initial excitation pulse.

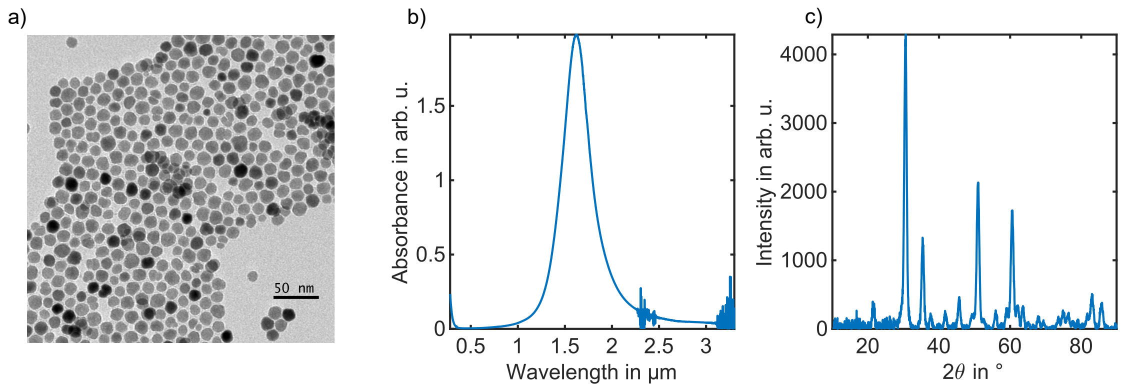

Figure 1: SI. a) Representative TEM image of the ITO nanocrystals batch employed for film deposition. b) Absorbance spectrum of 10 µL of a 1 $\frac{\mathrm{mg}}{\mathrm{mL}}$ dispersion diluted in 700 µL of hexane. c) XRD pattern of the resulting ITO film, displaying the characteristic diffraction peaks of crystalline ITO.

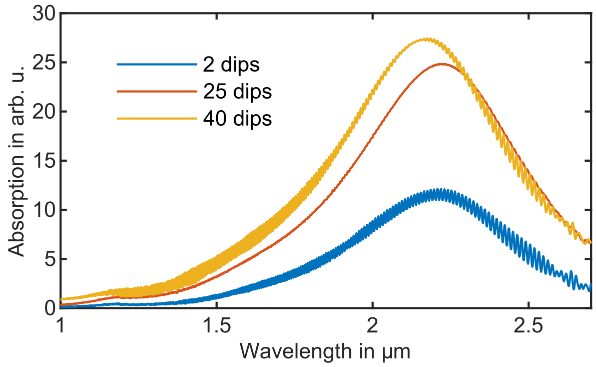

Figure 2: SI. Absorption spectrum of the nanocrystal films at three different thicknesses measured by PerkinElmer Lambda 950 UV/VIS wavelength scanning spectrometer.

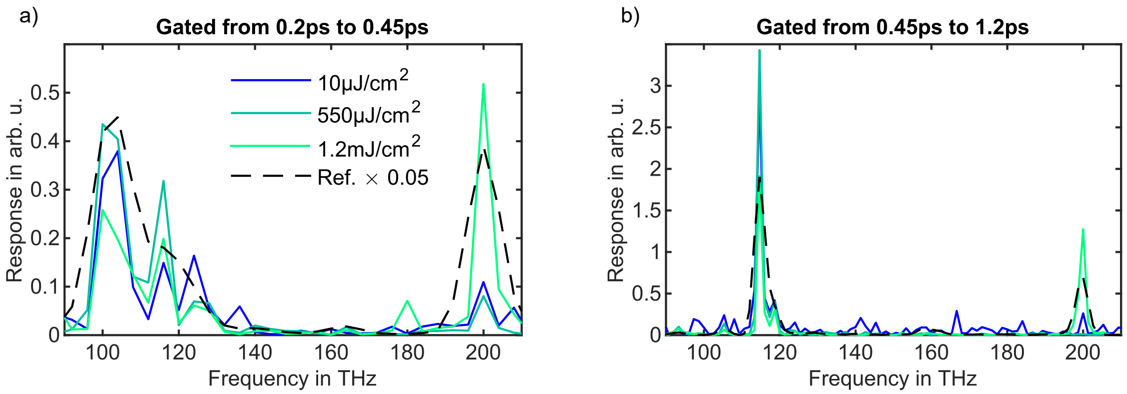

Figure 3: SI. Spectral Intensity response of the sample calculated within the time windows a) 0.2 ps to 0.45 ps and b) 0.45 ps to 1.2 ps. The dashed line represents the spectrum of the reference field. Resonances of atmospheric water molecules appear at 120 THz and 160 THz, while the absorption at 200 THz originates from the dielectric beam splitter in the beam path. The response of the nanocrystals at different fluences is imprinted on these long-lived spectral fingerprints of atmospheric molecules and can be exploited to probe the transient response of the sample on longer time scales.

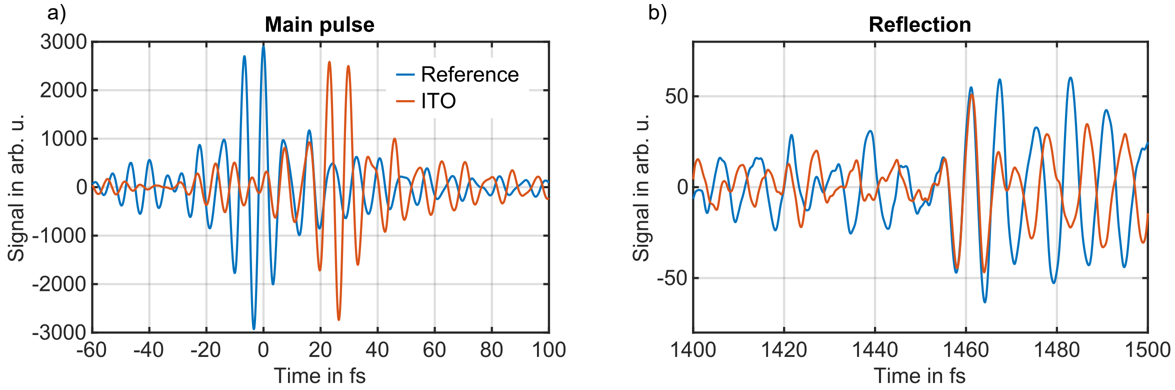

Figure 4: SI. The reflection of the excitation pulse at the substrate–air interface appears in the measured electric field at a temporal delay of 1.46 ps. For the sample containing ITO nanocrystals, this reflection occurs 25 fs earlier than for the pure substrate. Panels (a) and (b) illustrate the relative temporal separation of the excitation pulses, aligned such that their reflections at 1.46 ps overlap in the measured electric fields.

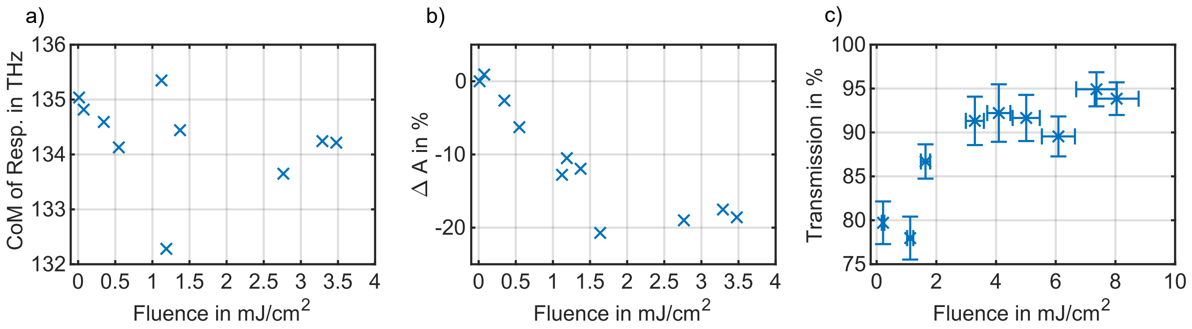

Figure 5: SI. a) Weighted mean frequency of the response. It can be seen that the red shift of the absorption disappears when the reversibility is compromised. b) Modulation depth (change in attenuation), obtained from the integrated spectral intensity of the measured field with and without ITO. The modulation depth remains constant at higher fluences. c) The measured transmitted power at various fluences by a thermal powermeter.

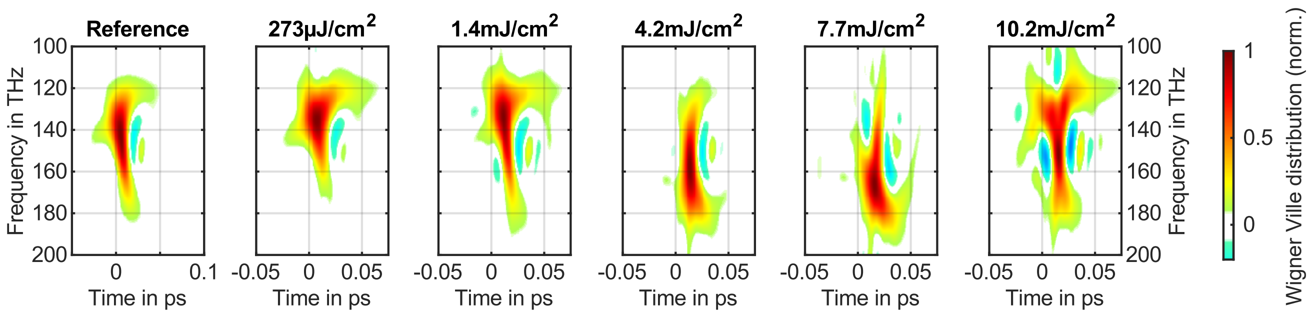

Figure 6: SI. Normalized Wigner–Ville distribution (WVD) of the substrate and sample responses at fluences between 273 $\frac{\mu J}{cm^2}$ and 10.2 $\frac{mJ}{cm^2}$. At low fluence, the response spectrum is dominated by the LSPR, whereas at higher fluences, the LSPR feature progressively bleaches. At 4.2 $\frac{mJ}{cm^2}$ the WVD exhibits a pronounced blue shift. At 7.7 $\frac{mJ}{cm^2}$ the WVD closely resembles that of the reference, indicating a spectrally uniform intensity loss.

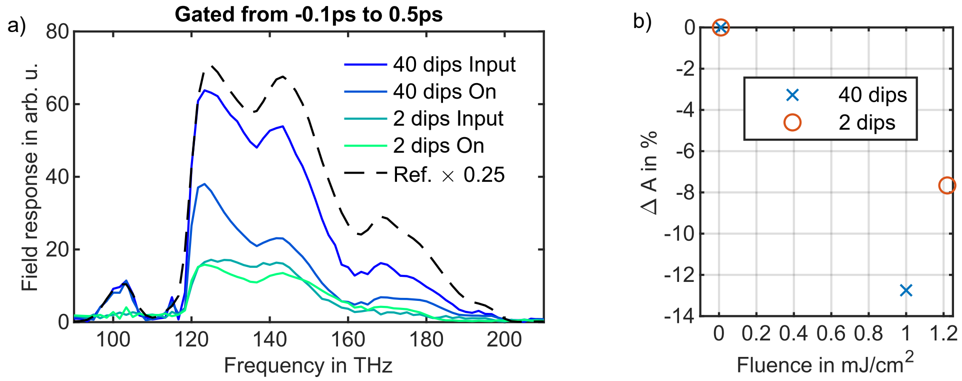

Figure 7: SI. a) Optical switching of a 2-dip sample and a 40-dip sample illuminated at similar fluences. b) Modulation depth (change in attenuation), obtained from the integrated spectral intensity of the measured field with and without ITO for two different thicknesses. The 1-dip sample shows 1.8 times lower modulation compared to the 40-dip sample at similar fluences.

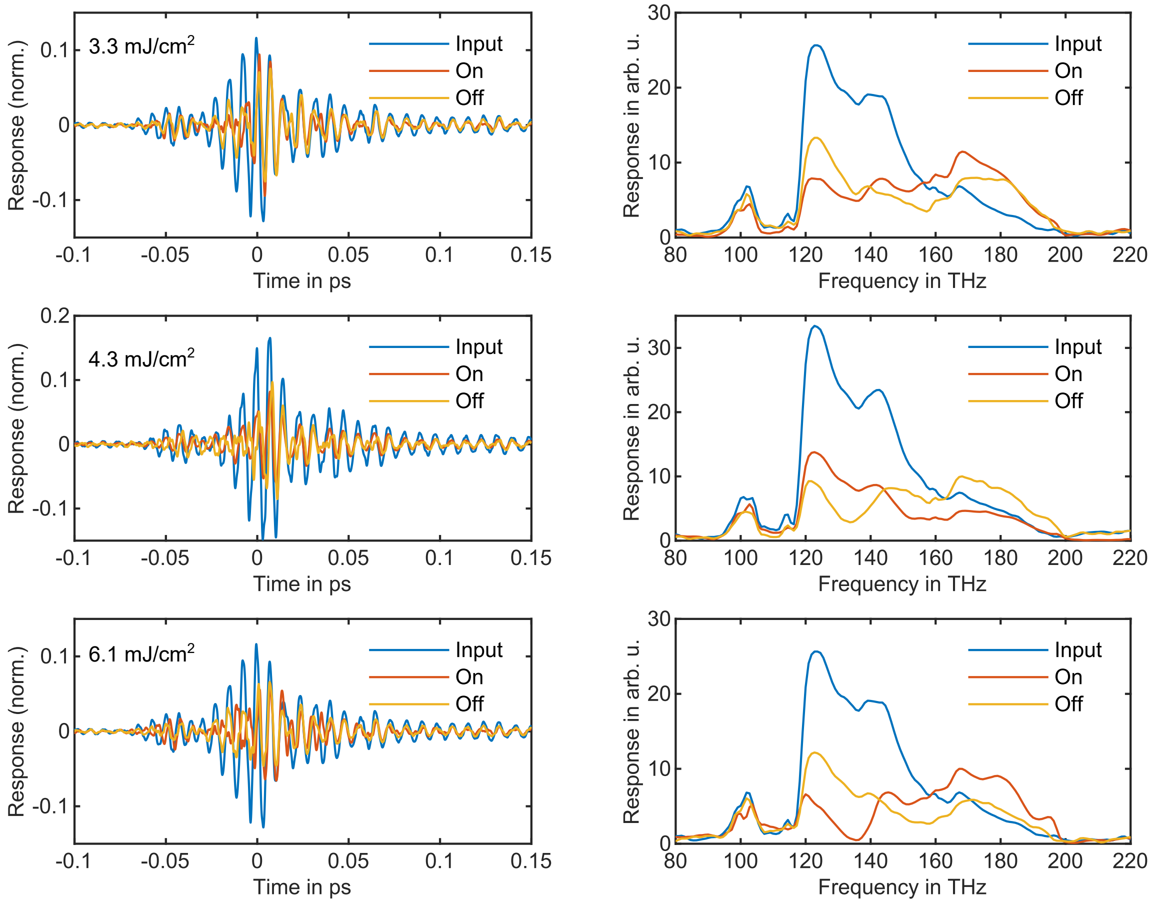

Figure 8: SI. Nonlinearity and reproducibility of the optical switching of the ITO nanocrystals at fluences beyond 3.3 $\frac{\mathrm{mJ}}{\mathrm{cm}^2}$. It is seen that the switch becomes fully irreversible at these fluences.
